# New quantitative trait locus (QTLs) and candidate genes associated with the grape berry color trait identified based on a high-density genetic map

**DOI:** 10.1186/s12870-020-02517-x

**Published:** 2020-06-30

**Authors:** Lei Sun, Shenchang Li, Jianfu Jiang, Xiaoping Tang, Xiucai Fan, Ying Zhang, Jihong Liu, Chonghuai Liu

**Affiliations:** 1 Zhengzhou Fruit Research Institute, Chinese Academy of Agricultural Sciences, Zhengzhou, China; 2 Key Laboratory of Horticultural Plant Biology (MOE), College of Horticulture and Forestry Sciences, Huazhong Agricultural University, Wuhan, China; 3 Pomology Institute, Shanxi Academy of Agricultural Sciences, Taiyuan, China

**Keywords:** Grape, Berry color, Anthocyanin, High-density genetic map, Quantitative trait locus (QTL), Candidate genes

## Abstract

**Background:**

Berry color is an important trait in grapes and is mainly determined by the anthocyanin content and composition. To further explore the coloring mechanism of grape berries, the F1 population of *Vitis vinifera* ‘Red Globe’ × ‘Muscat Hamburg’ was used to map the color locus, and transcriptome analysis was performed to assist in screening candidate genes.

**Results:**

A total of 438,407 high-quality single-nucleotide polymorphisms (SNPs) were obtained from whole-genome resequencing (WGS) of the population, and 27,454 SNPs were selected to construct a high-density genetic map. The selected SNPs were clustered into 19 linkage groups (LGs) spanning a genetic distance of 1442.638 cM. Berry color was evaluated by color grade, chromatic aberration, total anthocyanin content and anthocyanin composition. The Pearson correlation coefficients of these phenotypes in 2017 and 2018 were significant at the 0.01 level. The major color locus of *MYBA1* and *MYBA2* on LG2 was identified, explaining between 26 and 63.6% of all phenotypic variance. Furthermore, 9 additional QTLs with smaller effects were detected on Chr2, Chr4, Chr6, Chr11 and Chr17. Combined with the gene annotation and RNA-seq data, multiple new candidate genes were selected from the above QTLs.

**Conclusion:**

These results indicated that grape berry color is a quantitative trait controlled by a major color locus and multiple minor loci. Though the major color locus was consistent with previous studies, several minor QTLs and candidate genes associated with grape berry color and anthocyanin accumulation were identified in this study. And the specific regulatory mechanism still needs to be further explored.

## Background

Grapevines are an important economic crop, and berry color is an important trait in grapes. It is well known that the color of grape berries depends on the anthocyanins in the skin [1]. Anthocyanins are synthesized in the endoplasmic reticulum via the flavonoid pathways and are then transported to vacuoles for preservation by a series of transporters (Fig. S1) [2–4].

The flavonoid pathway is regulated by a transcription complex composed of MYB, bHLH and WD40 (MBW)[5, 6]. There is no doubt that anthocyanin biosynthesis is also regulated by the MBW transcription complex [7, 8]. The glycosylation catalyzed by UDP-glucose:anthocyanidin:flavonoid glucosyltransferase (UFGT) is the key step of anthocyanin biosynthesis. In grapes, the expression of *UFGT* is transcriptionally regulated by a series of MYB transcription factors (TFs) on chromosome 2 [3]. MYBA1 and MYBA2 are key TFs that participate in the regulation of anthocyanin biosynthesis by regulating the expression of *UFGT* in grapes. In *V. vinifera*, a retrotransposon (*Gret1*) was detected in the 5′-flanking region of the *VvMYBA1* gene, which formed a nonfunctional allele *VvMYBA1a* and affected its function by preventing its normal expression [9, 10]. Furthermore, the base mutation in the coding region of the *VvMYBA2* gene leads to the early termination of its translation and thus forms the nonfunctional allele *VvMYBA2w*. White-skin grapes are caused by mutation of both the *VvMYBA1* and *VvMYBA2* genes [11, 12], which well explained the origin of white-skin grapes. However, the color of grape berry skin is a complex trait with many intermediate coloring types. How do so many coloring types come about? Some studies have shown that the color of grape berries is closely related to the haplotype composition of *MYBA* genes [13, 14]. However, this cannot fully explain the color separation of grapes.

Anthocyanin biosynthesis is a complex metabolic process that is regulated by a network comprising a series of regulators. Some regulators have been identified in many other plants and grapes. In *Arabidopsis*, the MYB-Like 2 (MYBL2) protein [15, 16], Jasmonate ZIM-domain (JAZ) [17] and Squamosa Promoter Binding Protein-like (SPL) [18] were demonstrated to inhibit the action of the MBW complex by interacting with its corresponding members, thereby inhibiting anthocyanin synthesis. On the other hand, studies showed that MYBD, the ELONGATED HYPOCOTYL 5 (HY5) protein, DELLA and some other proteins could promote anthocyanin biosynthesis by inhibiting the action of these repressors [19–21]. Recently, three new R2R3-MYB genes (ASR1, ASR2, ASR3) were identified and shown to be involved in anthocyanin synthesis in *Petunia* [22]. In apple, a series of *MYB* genes (*MdMYB1/MdMYB10*, *MdMYB9*, *MdMYB11*) [23–25], *bHLH* genes (*MdbHLH3* and *MdMYC2*) [26, 27] and the WD40 gene (*MdTTG1*) [28] were identified to participate in the anthocyanin synthesis regulation. In recent years, in addition to MYBA1 and MYBA2, other anthocyanin synthesis regulators have been identified in grapes. Two MYB TFs (MYBC2-L1 and VvMYB4-Like) were cloned and shown to play negative regulatory roles in anthocyanin biosynthesis in grape berry skin [29, 30]. Two miRNAs (miRNA828 and miRNA858) regulate the expression of the *MYB* gene to promote anthocyanin accumulation in grapes [31]. These results show that grape berry color is a complicated trait that is regulated by multiple loci and multiple genes.

Quantitative trait locus (QTL) mapping is an effective way to find functional loci and genes. The construction of a high-quality genetic linkage map is the basis of QTL mapping. The earliest genetic maps were constructed based on RAPD and AFLP markers [32, 33]. A large number of genetic maps have been constructed for grapes based on traditional DNA markers, such as RAPD, AFLP, SRAP and SSR markers [34–38]. However, the number of these DNA markers is limited, so it is difficult to construct a high-density genetic map with these traditional markers. Subsequently, the publication of the full grape genome [39] and the rapid development of sequencing technology promoted the development and wide utilization of single-nucleotide polymorphism (SNP) markers [40]. SNPs are the most abundant and stable genetic variations in the genome and are ideal markers for the construction of high-density genetic maps. In recent years, multiple QTLs and candidate genes for related traits have been identified using high-density genetic maps constructed based on SNP markers in grapes [41–47]. Therefore, high-density genetic maps show obvious advantages in QTL mapping.

In this study, the whole-genome resequencing (WGS) strategy was used to identify SNPs for constructing high-density genetic maps in a population of *V. vinifera* ‘Red Globe’ and ‘Muscat Hamburg’. QTL mapping was carried out from the perspectives of apparent color, anthocyanin content and anthocyanin composition. Multiple QTLs related to the berry color trait were identified based on the high-density genetic map. After combining the results of QTLs and transcriptome analysis, several candidate genes were selected that may be related to anthocyanin accumulation and berry color determination. This provides a reference for further studies on the regulation of grape berry color.

## Results

### The WGS data and SNP markers

After WGS, a total of 715.24 Gb and 42.72 Gb of high-quality clean reads were obtained from the 95 individuals in the F1 population and the parents, respectively. And the clean reads have been submitted to SRA database of NCBI (Accession ID: PRJNA589353). The average GC content was 37.12%. The average coverage rate of clean reads in the genome was 91.70%, and the average mapping rate was 96.36% (Table S1). This indicates that the sequencing reads were distributed randomly across the genome. The average coverage depths of the markers were 43.78-fold for the female parent, 50.55-fold for the male parent, and 16.62-fold for their progeny (Table S1).

Through rigorous filtering (depth > =10, quality value > = 40, and SNPs with a miss rate of more than 20% or a nondimorphic type were filtered), a total of high-quality 438,407 SNPs were obtained from the population. After further filtration, 27,454 SNPs were used to construct the high-density genetic map.

### Construction of the genetic map

The map of the female parent, ‘Red Globe’, anchored 12,325 SNPs in 733 bin markers across 19 chromosomes (Chrs), with genetic distances of 1271.352 cM. The average marker distance was 1.734 cM. The average number of SNPs in ‘Red Globe’ was 770 per Chr. The map of the male parent, ‘Muscat Hamburg’, anchored 15,404 SNPs in 929 bin markers across 19 Chrs, with genetic distances of 1614.853 cM. The average marker distance was 1.738 cM. The average number of SNPs in ‘Muscat Hamburg’ was 811 per Chr (Table S2).

**Fig. 1:**
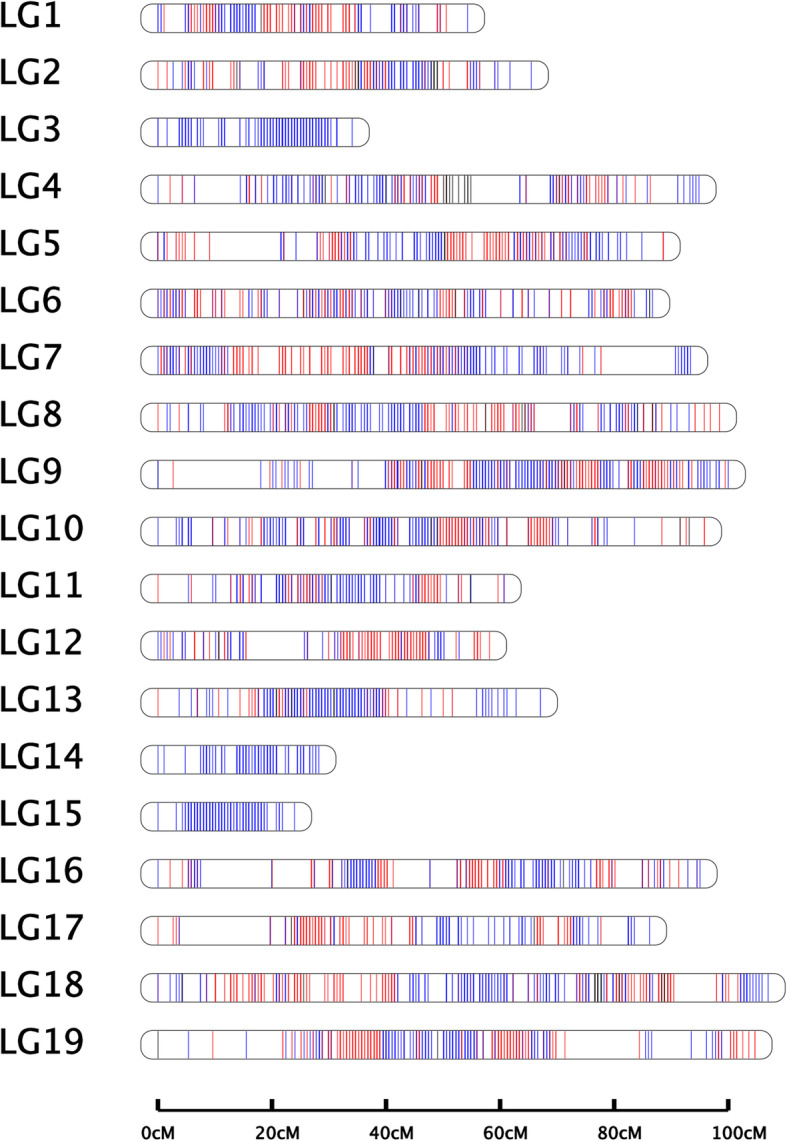
Integrated genetic map of ‘Red Globe’ × ‘Muscat Hamburg’. The red, blue and black lines refer to the markers from the female parent map, the markers from the male parent map and the sites at which both parents are heterozygous, respectively

The integrated genetic map was constructed based on 27,454 SNPs in 1554 bin markers, which clustered in 19 linkage groups (LGs) and covered 1442.638 cM (Fig. 1; Table 1). The average marker distance was 0.928 cM. The average number of SNPs and bin markers per LG were 1445 and 82, respectively. The genetic distance of the LGs ranged from 34.051 cM (LG3) to 107.001 cM (LG18), with an average distance of 75.928 cM per LG. The “max gap” of the LGs ranged from 2.662 cM (LG3) to 15.945 cM (LG17). The average percentage of “Gap < 5 cM” was 99.928% (Table 1). The Spearman correlation coefficients of collinearity analysis between genetic and physical map ranged from 0.87 (LG18) to 0.97 (LG4 and LG12), the average Spearman of 19 LGs was 0.93 (Fig. S2; Table S3). The results showed that 19 LGs have high levels of genetic collinearity with the physical map.

**Table 1:** The distribution of markers in the integrated genetic maps

Linkage groups	Genetic distance (cM)	SNP markers	Bin markers	Average distance (cM)	Max gap (cM)	Gap < 5 cM (%)
LG1	54.285	813	65	0.835	3.731	100
LG2	65.459	666	72	0.909	3.730	100
LG3	34.051	1449	44	0.774	2.662	100
LG4	94.870	599	91	1.043	8.595	99.666
LG5	88.580	1777	93	0.952	12.488	99.944
LG6	86.732	692	102	0.850	3.196	100
LG7	93.399	979	104	0.898	13.055	99.898
LG8	98.463	836	118	0.834	6.418	99.880
LG9	100.002	3567	118	0.847	15.359	99.944
LG10	95.823	2691	101	0.949	4.802	100
LG11	60.691	812	65	0.934	5.339	99.877
LG12	58.131	1051	62	0.938	10.248	99.905
LG13	67.061	2158	72	0.931	4.266	100
LG14	28.204	502	34	0.830	3.730	100
LG15	23.943	1068	35	0.684	3.196	100
LG16	95.054	2988	83	1.145	12.487	99.900
LG17	86.200	868	70	1.231	15.945	99.885
LG18	107.001	1155	124	0.863	7.502	99.913
LG19	104.689	2783	101	1.037	13.055	99.820
Total	1442.638	27,454	1554	0.928	–	–
Average	75.928	1444.947	81.789	0.920	7.884	99.928

### Phenotypic analysis of berry color

**Fig. 2:**
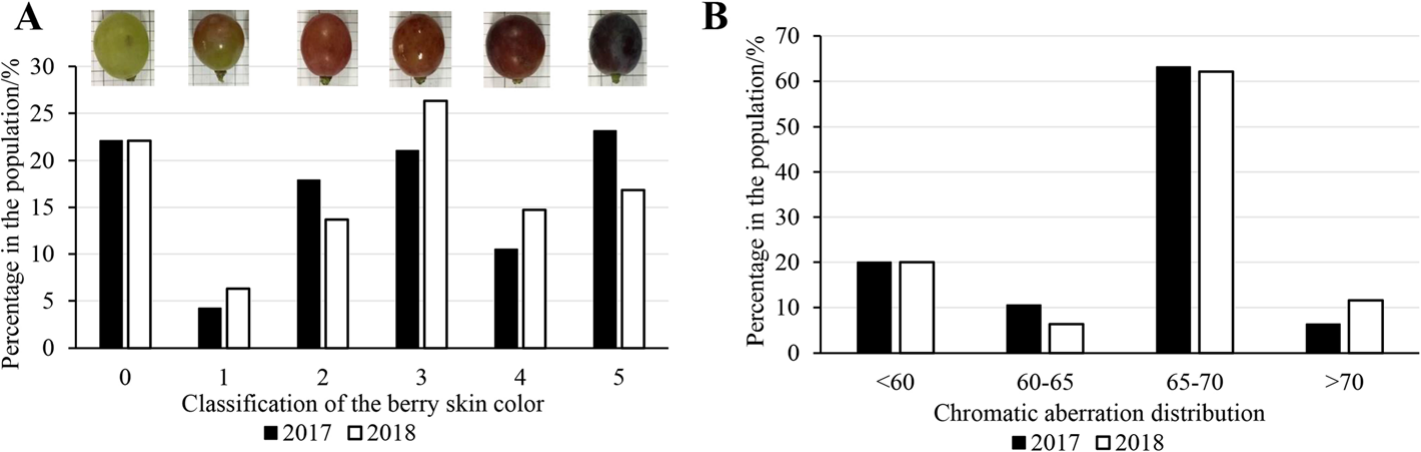
Distribution of color grade (CG) and chromatic aberration (CA) among progeny of ‘Red Globe’ X ‘Muscat Hamburg’ in 2017 and 2018. **a**, Distribution of color grade. **b**, Distribution of chromatic aberration

In 2017 and 2018, the berry coloration of the population showed obvious trait separation from yellow-green to purple-black, which was graded from level 0 to level 5 (Fig. 2a). In the population, the offspring of 21 yellow-green coloring types were very stable. In addition to the extreme coloring types, yellow-green and purplish-black (level 0 and level 5), intermediate coloring types (levels 1–4) accounted for approximately 60% of the population (Fig. 2a). The Pearson correlation coefficient of color grade between 2017 and 2018 was 0.916 (Table 2).

**Table 2:** Pearson correlation coefficients of color grade and chromatic aberration

Pearson correlation	Color grade		Chromatic aberration	
	2017	2018	2017	2018
2017	1	0.916^**^	1	0.911^**^
2018		1		1

^**^The correlation is significant at the 0.01 level

In addition, the coloration of the population was also evaluated by a colorimeter. This represented a way to quantify the coloration of grape berry skin. In the population, the chromatism value ranged from 52.37 to 71.59 in 2017 and from 52.16 to 74.33 in 2018. The chromatism values of all yellow-green coloring types were below 60, and those of the purple-black coloring types were approximately 70. The chromatism values of most offspring ranged from 65 to 70, with these values accounting for more than 60% of the population (Fig. 2b). The Pearson correlation coefficient of chromatic aberration between 2017 and 2018 was 0.911 (Table 2).

**Fig. 3:**
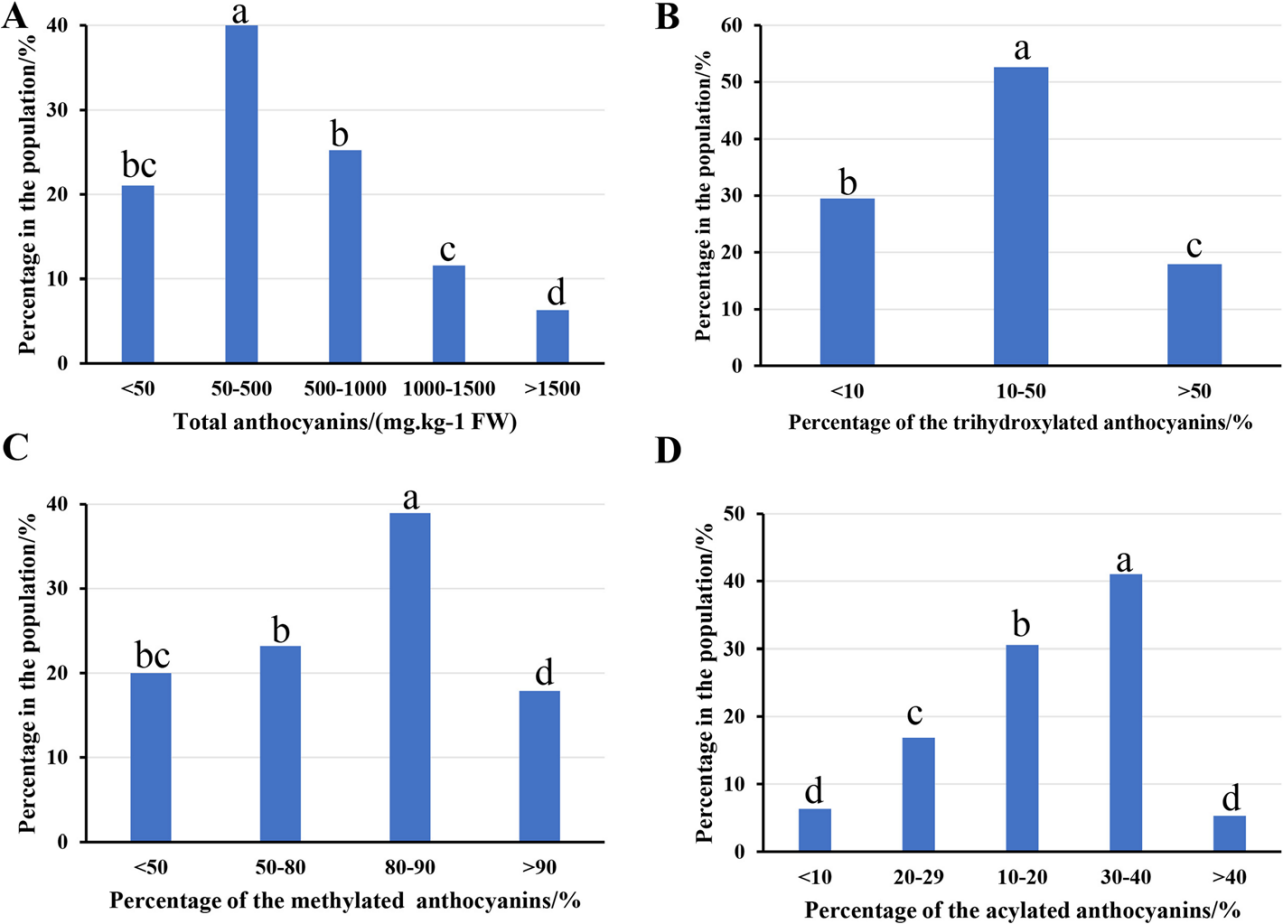
Distribution of total anthocyanins (TA) and the proportions of trihydroxylated anthocyanins (DC), methylated anthocyanins (MC) and acylated anthocyanins (AC) among progeny of the population. **a**, **b**, **c**, and **d** refer to the distribution of TA, DC, MC and AC, respectively

To better evaluate the berry color trait, the components and contents of anthocyanins in berry skin in the population were determined by UPLC-MS. The total anthocyanin content in the berry skin varied from 1.43 mg/kg (FW) to 1869.99 mg/kg (FW) in the population. The total anthocyanin content of most offspring ranged from 50 mg/kg (FW) to 1000 mg/kg (FW), with these values accounting for more than 65% of the population (Fig. 3a). Regarding the anthocyanin components, a total of 12 kinds of anthocyanins were detected in the berry skin within the population (Table S4). In this study, both dihydroxylated (cyanidin-based and peonidin-based) and trihydroxylated (delphinidin-based, petunidin-based and malvidin-based) anthocyanins were detected in the berry skin within the population. Although the percentage of trihydroxylated anthocyanins in all offspring was less than 50%, they also showed obvious separation. The trihydroxylated anthocyanins accounted for between 10 and 50% of anthocyanins in more than 50% of the offspring (Fig. 3b). Methylation and acylation of anthocyanins greatly enrich the variety of anthocyanins in grapes. Peonidin-based anthocyanins are formed on the basis of cyanidin-based anthocyanin methylation, and petunidin-based and malvidin-based anthocyanins are formed on the basis of delphinidin-based anthocyanin methylation. In this population, methylated anthocyanins accounted for a large proportion of anthocyanins, and in 80% of the offspring, they accounted for more than 50% of anthocyanins. In addition, in more than half of offspring, methylated anthocyanins accounted for more than 80% of anthocyanins (Fig. 3c). Acylated anthocyanins were also detected in the offspring. However, acylated anthocyanins were present in less than 50% of all the offspring, and most of these anthocyanins were distributed in 10 to 40% of offspring (Fig. 3d).

### QTL analysis

**Fig. 4:**
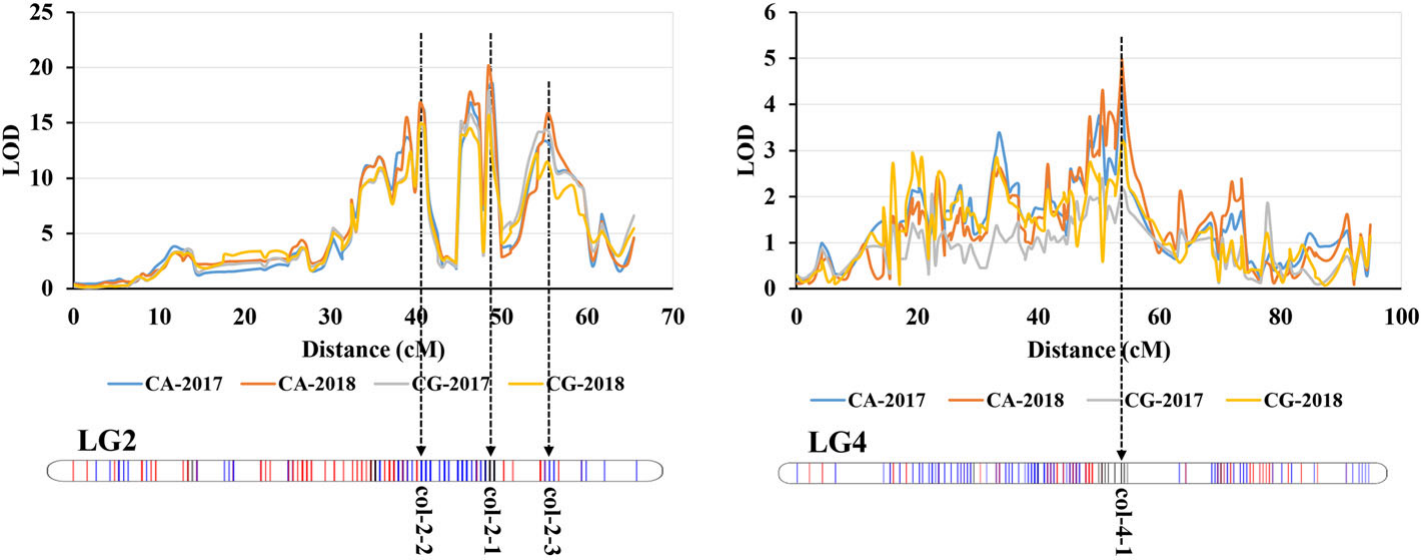
The QTL localization for color grade (CG) and chromatic aberration (CA) in 2017 and 2018 on LG2 and LG4

Based on the high-density genetic map, the QTLs associated with berry color traits were identified. Regarding color grade (CG) and chromatic aberration (CA), 4 loci were identified that were consistent in 2017 and 2018 (Fig. 4). Among these loci, 3 loci (col-2-1, col-2-2 and col-2-3) on Chr2 were identified based on the phenotype of both CG and CA. Locus col-2-1 explained 62.5% of the phenotypic variation in CA, with a maximum LOD score of 20.01 in 2018 (Table 3). This was obviously a major QTL for the berry color trait. In addition, one locus, col-4-1, was detected on Chr4 and explained 22.2 and 19% of the phenotypic variation for CA, with LOD scores of 4.8 and 4.31 in 2017 and 2018, respectively (Table 3).

**Table 3:** QTLs for CG and CA in 2017 and 2018

Phenotype	QTLs	Chr	Position (cM)	Left_markers	Right_markers	LOD	PVE
CA-2017	col-2-1	2	45.231–48.955	2_12325676	2_16199608	18.48	62.0
	col-2-2	2	37.252–41.507	2_4327749	2_7978280	16.52	57.9
	col-2-3	2	54.278–59.600	2_18472872	2_18578300	13.30	50.2
	col-4-1	4	48.481–54.332	4_7387933	4_9951732	4.80	22.2
CA-2018	col-2-1	2	45.231–48.955	2_12325676	2_16199608	20.01	62.5
	col-2-2	2	37.252–41.507	2_4327749	2_7978280	16.73	56.0
	col-2-3	2	54.278–59.600	2_18472872	2_18578300	15.80	53.9
	col-4-1	4	48.481–54.864	4_7387933	4_10014514	4.31	19.0
CG-2017	col-2-1	2	45.231–48.955	2_12325676	2_16199608	17.55	57.7
	col-2-2	2	37.784–41.507	2_4810938	2_7978280	14.55	51.0
	col-2-3	2	54.278–59.600	2_18472872	2_18578300	14.34	50.5
CG-2018	col-2-1	2	45.231–48.955	2_12325676	2_16199608	15.42	53.0
	col-2-2	2	37.784–41.507	2_4810938	2_7978280	14.83	51.6
	col-2-3	2	54.278–59.600	2_18472872	2_18578300	11.50	43.1

*CA* chromatic aberration, *CG* color grade

**Fig. 5:**
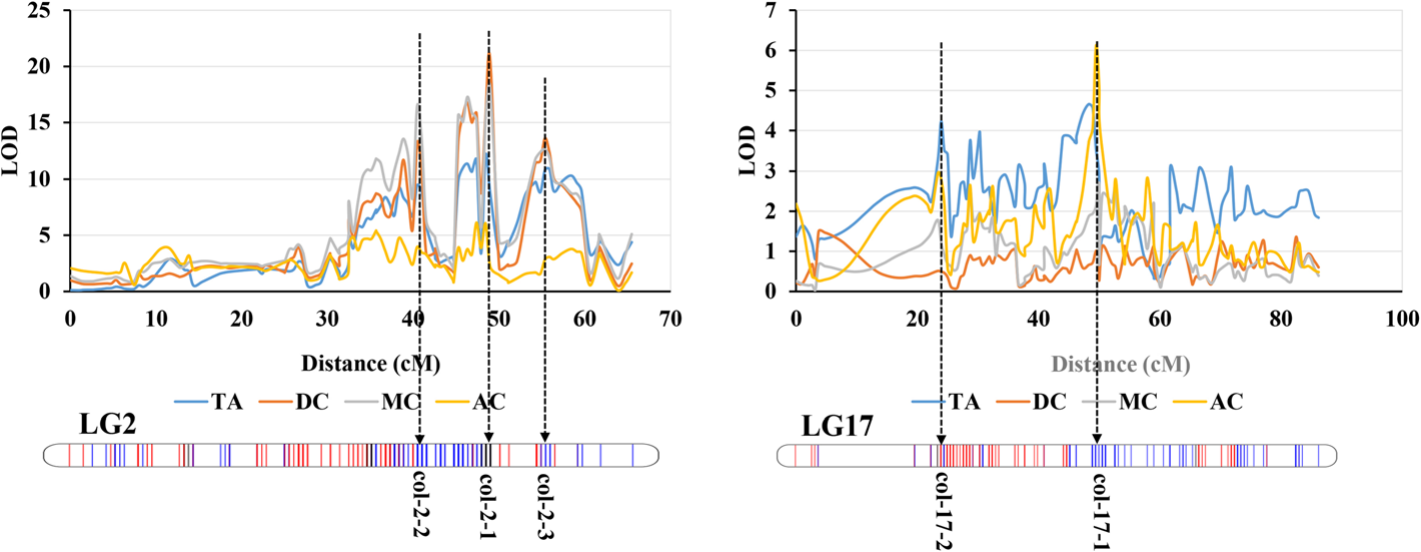
The QTL localization for total anthocyanins (TA) and the proportions of trihydroxylated anthocyanins (DC), methylated anthocyanins (MC) and acylated anthocyanins (AC)

The color of grape berries is closely related to the anthocyanin content and components in the berry skin. The phenotypes of total anthocyanins (TA) and the proportions of trihydroxylated anthocyanins (DC), methylated anthocyanins (MC) and acylated anthocyanins (AC) were used to perform QTL mapping. This finding was consistent with the results of CG and CA, and the major QTL col-2-1 was identified for all four phenotypes. On Chr2, loci col-2-2 and col-2-3 were also identified based on the phenotypes of TA, DC and MC (Fig. 5; Table 4). In addition, two other loci (col-17-1 and col-17-2) on Chr17 were identified based on TA and AC. The locus col-17-1 was associated with both TA and AC, and the locus col-17-2 was associated with only TA (Fig. 5; Table 4). Furthermore, two loci (col-4-1 and col-4-2) on Chr4, one locus (col-6-1) on Chr6 and two loci (col-11-1 and col-11-2) on Chr11 were identified based on these four phenotypes (Fig. S3; Table 4).

**Table 4:** QTLs associated with anthocyanin content and composition

Phenotype	QTLs	Chr	Position (cM)	Left markers	Right markers	LOD	PVE
TA	col-2-1	2	45.763–48.955	2_13368581	2_16199608	12.07	44.6
	col-2-2	2	38.316–40.975	2_4810938	2_7480841	9.45	37.1
	col-2-3	2	54.278–59.600	2_18472872	2_18578300	10.87	41.3
	col-17-1	17	46.278–49.472	17_8320296	17_10186338	4.56	20.0
	col-17-2	17	23.929–24.993	17_15811325	17_17122672	4.23	18.7
	col-11-2	11	44.184–54.825	11_1209685	11_3614745	3.75	16.8
DC	col-2-1	2	45.231–48.955	2_12325676	2_16199608	20.62	63.6
	col-2-2	2	37.784–40.975	2_4810938	2_7480841	13.17	47.5
	col-2-3	2	54.278–59.600	2_18472872	2_18578300	13.58	48.6
	col-4-1	4	50.077–54.864	4_8364168	4_9951732	2.96	13.5
MC	col-2-1	2	45.231–48.955	2_12325676	2_16199608	18.18	59.0
	col-2-2	2	37.784–41.507	2_4810938	2_7978280	16.49	55.4
	col-2-3	2	54.278–59.600	2_18472872	2_18578300	12.68	46.3
	col-4-1	4	50.077–54.864	4_8364168	4_9951732	3.23	14.6
	col-6-1	6	68.635	6_3497067	6_4286240	2.85	13.0
AC	col-2-1	2	46.295–48.423	2_13046874	2_15989982	6.13	26.0
	col-17-1	17	48.94–50.536	17_9980211	17_10653159	6.11	25.9
	col-4-2	4	63.459–64.523	4_3339314	4_5265763	3.62	16.2
	col-6-1	6	68.639	6_3497067	6_4286240	2.77	12.7
	col-11-1	11	27.693–29.820	11_9515984	11_11099209	3.79	17.0

TA refers to total anthocyanins; DC, MC and AC refer to the proportions of trihydroxylated anthocyanins, methylated anthocyanins and acylated anthocyanins, respectively

### Gene expression analysis in QTL regions and selection of candidate genes

**Fig. 6:**
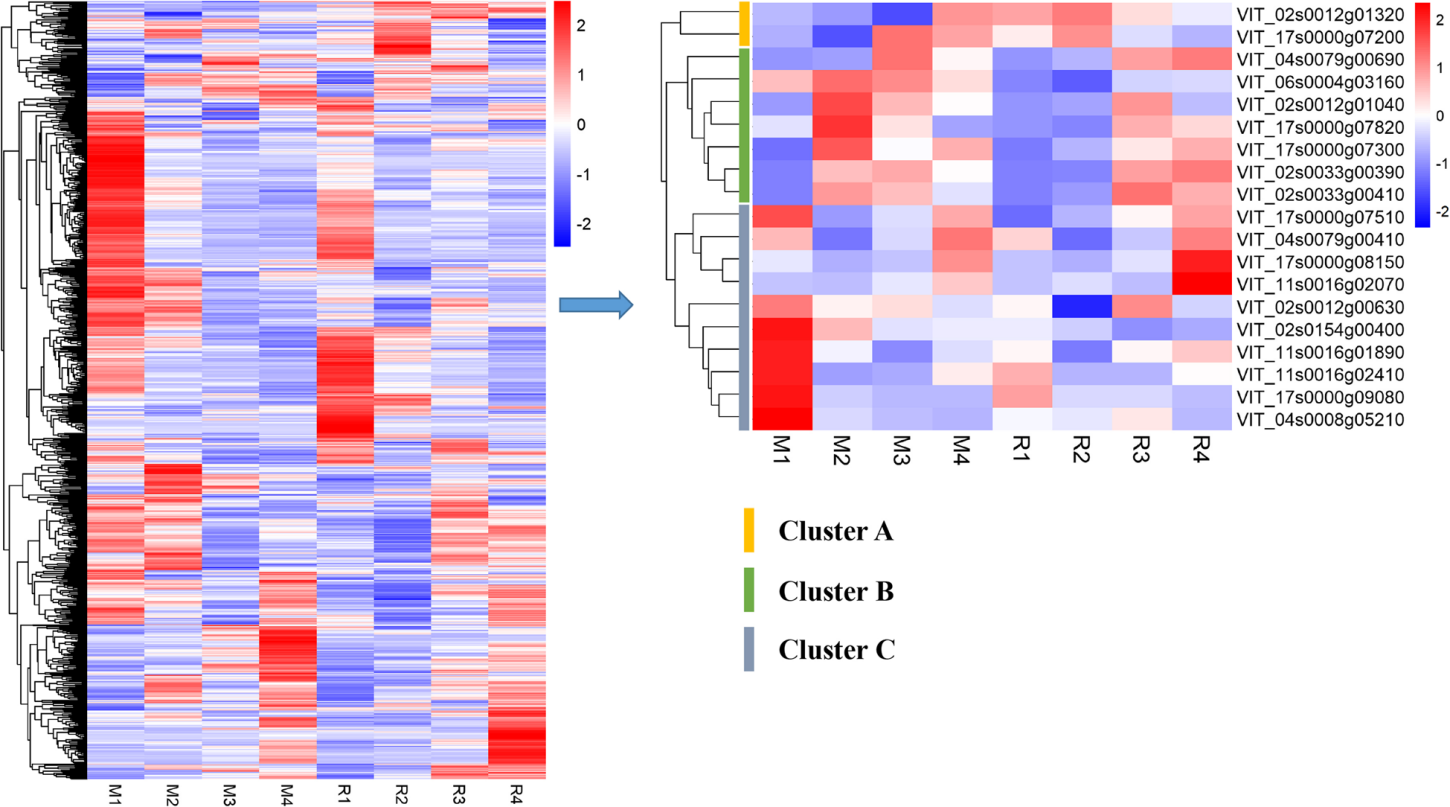
The expression patterns of all genes and candidate genes in the QTL regions. M1, M2, M3, and M4 and R1, R2, R3, and R4 refer to the four berry development stages of ‘Muscat Hamburg’ and ‘Red Globe’, respectively. The block colored from blue to red indicates a gradual increase in gene expression level

According to the annotation data for the grape reference genome (PN40024 assembly 12X), a total of 1056 genes were identified in the above QTL regions. The expression of these genes was analyzed based on transcriptome data from the parents ‘Red Globe’ and ‘Muscat Hamburg’ at 4 different berry developmental stages. The clean data of RNA-Seq have been submitted to SRA database of NCBI (Accession ID: PRJNA627817). The results of qRT-PCR analysis showed that the expression patterns of 20 genes related to anthocyanin accumulation in grape berry skin were highly consistent with the results of RNA-Seq, indicating that the data of RNA-Seq was reliable (Fig. S4). In the above QTL regions, 748 genes expressed in the berry skin of the parents. The expression levels and annotation information of these genes are shown in Table S5. A total of 19 candidate genes were selected based on the expression pattern analysis and annotation information (Fig. 6; Table 5). Two *MYB* genes (*MYBA1* and *MYBA2*) were located in the major locus col-2-1. Furthermore, 4 genes in locus col-2-2, 6 genes in locus col-17-1, 2 genes in locus col-4-1, 1 gene in locus col-4-2, 1 gene in locus col-6-1, and 3 genes in locus col-11-2 were identified as candidate genes that may be related to the regulation of grape berry skin (Table 5). These 19 genes showed three expression patterns (cluster A, cluster B and cluster C), and most of these candidate genes were expressed in the patterns of cluster B and cluster C (Fig. 6). As the berries developed, the expression levels of the genes in cluster B first increased and then decreased. In contrast, the expression levels of the genes in cluster C first decreased and then increased.

**Table 5:** Candidate genes were screened from the QTL regions

QTLs	Gene ID	FPKM Value								Annotation
		M1	M2	M3	M4	R1	R2	R3	R4	
col-2-1	VIT_02s0033g00390	0.00	35.40	39.36	24.18	0.00	1.45	41.25	48.05	MYBA2
	VIT_02s0033g00410	0.11	176.28	146.15	78.33	0.01	20.16	204.54	159.69	MYBA1
col-2-2	VIT_02s0154g00400	85.86	59.40	44.25	45.74	45.24	41.02	30.45	35.03	GRAS
	VIT_02s0012g00630	22.56	18.46	19.15	16.81	18.36	10.22	22.13	16.38	MYB
	VIT_02s0012g01040	2.19	29.44	18.33	12.11	1.61	3.37	21.91	5.48	NAC
	VIT_02s0012g01320	52.25	48.77	40.05	71.81	70.10	74.29	63.87	57.63	bHLH
col-17-1	VIT_17s0000g07200	150.04	83.54	314.18	278.86	224.36	292.87	185.42	154.32	F3’H
	VIT_17s0000g07300	96.00	151.94	120.97	135.43	97.58	107.45	125.44	135.29	H^+^-ATPase
	VIT_17s0000g07510	190.96	80.01	107.42	154.60	62.70	91.82	123.71	156.61	MYB
	VIT_17s0000g07820	80.06	142.61	95.25	62.96	58.91	55.41	109.85	97.68	LIM
	VIT_17s0000g08150	6.96	2.00	3.83	18.75	3.89	1.91	6.45	28.34	bHLH
	VIT_17s0000g09080	16.64	1.22	0.25	0.16	9.05	1.99	1.89	0.62	MYB
col-4-1	VIT_04s0079g00410	57.69	18.05	36.25	69.99	52.77	16.17	34.16	68.21	MYB
	VIT_04s0079g00690	0.45	95.32	2385.51	1093.51	0.12	313.27	1928.82	2290.31	GST
col-4-2	VIT_04s0008g05210	87.14	13.28	6.77	4.20	21.54	16.60	29.24	5.30	HY5
col-6-1	VIT_06s0004g03160	178.49	223.40	207.39	161.10	74.80	52.02	117.12	121.43	S1Fa-like
col-11-2	VIT_11s0016g01890	18.42	9.24	5.25	8.52	10.02	4.81	10.21	12.00	MYB
	VIT_11s0016g02070	0.25	0.05	1.42	3.99	0.31	1.04	0.17	10.37	bHLH
	VIT_11s0016g02410	10.20	0.63	0.91	4.03	5.82	1.13	1.21	3.56	MYB

## Discussion

Because berry color is an important trait in grapes, the regulatory mechanism of berry color has been a popular research topic for many years. Berry color in grapes is closely related to the anthocyanin content and composition in the berry skin [1]. Anthocyanin biosynthesis in grape berries is mainly regulated by a color locus on chr 2. The genotype of *MYBA1* and *MYBA2* genes at this color locus is closely related to grape berry color [9–13]. From the perspective of white and colored grapes, this is more of a quality trait. Mutation of both the *MYBA1* and *MYBA2* genes caused white-skin grapes, fully supporting this idea [11, 12]. However, for colored grapes, the berry color varies from the lightest pink to the darkest purple-black. The continuous distribution of grape berry coloration (Fig. 2) and anthocyanin content (Fig. 3a) was a typical characteristic of quantitative traits. Thus, it is difficult to fully explain the grape color separation based on this color locus.

In our study, the major QTL col-2-1 that contained the *MYBA* gene cluster on chromosome 2 was detected for all phenotypes associated with berry color, which explained between 26 and 63.6% of all phenotypic variance. This is consistent with previous studies. This also confirmed the reliability of our QTL mapping. Furthermore, a series of QTLs with smaller effects were identified on Chr2, Chr4, Chr6, Chr11 and Chr17, which were related to the anthocyanin content and composition of berry skin (Tables 3 and 4). To better explain the mechanism of berry color regulation, several studies have performed QTL mapping for grape berry color in recent years [48–52]. However, due to the low-density genetic map constructed by SSR markers, previous studies usually identified only the major color locus on chromosome 2. Costantini et al. [53] constructed a genetic map based on SSR markers and limited SNP markers to perform QTL mapping for grape color. The use of SNP markers greatly increased the density of the genetic map to improve the precision of QTL mapping. Thus, in addition to the major QTL on LG2, multiple other QTLs were identified on other LGs in the study of Costantini et al. [53]. In our study, a very high-density genetic map was constructed based on a large number of SNP markers developed by WGS (Fig. 1; Table 1). This provides a reliable basis for fine QTL localization to identify minor QTLs and resolve closely linked QTLs [54].

In addition to a good genetic map, accurate evaluation of the trait is also a key factor for QTL mapping. Grape berry color is a complex trait. To better evaluate the berry color trait, multiple methods were used in our research, including grade by subjective evaluation, measured by an accurate chromameter, and the anthocyanin content and composition detected by UPLC-MS. Thus, QTL mapping was performed based on different phenotypic descriptions of berry color. The Pearson correlation coefficients of CG and CA between 2017 and 2018 were 0.916 and 0.911 (Table 2), respectively. This indicated that grape berry color was a relatively stable trait and that genetic factors play a major role in its regulation.

For the subjective berry CG, the QTLs were detected only on LG2, including one major QTL, col-2-1, and two other QTLs (col-2-2 and col-2-3). In the major QTL col-2-1, the *MYBA1* (VIT_02s0033g00410) and *MYBA2* (VIT_02s0033g00390) genes were identified at this locus, which has been shown to play important roles in the regulation of anthocyanin synthesis [9–12]. There is no doubt that *MYBA1* and *MYBA2* are the major genes that regulate berry color of grape. On the QTL col-2-2, four transcription factor genes showed high expression levels in the parents, including one *GRAS* (VIT_02s0154g00400), one *MYB* (VIT_02s0012g00630), one *NAC* (VIT_02s0012g01040) and one *bHLH* (VIT_02s0012g01320) gene (Table 5). There is no doubt that a series of MYB and bHLH TFs have been confirmed to play important roles in color regulation [15, 16, 22, 24–27]. The candidate MYB and bHLH TFs at this locus may also be involved in color regulation. A study indicated that DELLA proteins participate in the regulation of anthocyanin biosynthesis in *Arabidopsis* [20]. As a member of the DELLA proteins, the candidate gene *GRAS* selected in this study may participate in the regulation of anthocyanin accumulation in grapes. NAC TFs have been shown to be involved in the regulation of anthocyanin synthesis in blood orange and blood-fleshed peach [55, 56]. In our study, the candidate gene annotated as a NAC transcription factor may perform a similar function in grapes. In addition to these three QTLs on LG2, another QTL, col-4-1, on LG4 was identified for CA (Table 3). Two candidate genes were selected at this locus, *MYB* (VIT_04s0079g00410) and *GST* (VIT_04s0079g00690). The *GST* gene showed a very high expression level in the parents and was identified to play an important role in anthocyanin transport in grapes [57]. Unfortunately, there were no suitable genes selected in the QTL col-2-3.

The color of grape berries is determined by the anthocyanin composition and content in the skin [1]. Thus, to more systematically evaluate the berry color trait, the anthocyanin composition and content of the progeny were detected by UPLS-MS. Then, QTL localization was carried out from the perspective of anthocyanin content and composition. Interestingly, the major QTL on LG2 was identified for all these phenotypes, and the LOD score ranged from 6.13 to 20.62 (Table 4). This result suggests that this major QTL not only affects the anthocyanin content but also regulates the components of anthocyanins. This result is consistent with previous studies [52, 53]. Azuma et al. [52] performed QTL analyses and showed that the color locus on LG2 was detected as the major QTL for the ratios of tri/di-hydroxylated and methylated/nonmethylated anthocyanins. In addition, they also found that a QTL on LG1 containing an *anthocyanin O-methyltransferase* (*AOMT*) gene played important roles in the synthesis of methylated anthocyanins. However, although the major color locus on LG2 was detected for the proportion of methylated anthocyanins, the QTL on LG1 was not detected in our study. Nevertheless, the QTL col-4-1, as mentioned above, was identified for the proportion of methylated and trihydroxylated anthocyanins and may be related to the regulation of anthocyanin composition. Furthermore, the major QTL on LG2 was detected for all kinds of anthocyanins by QTL mapping [53]. For the proportion of acylated anthocyanins, the very significant QTL col-17-1 was detected on LG17, the LOD score was 6.11, and 25.9% of the phenotypic variance was explained. This QTL was also detected for TA. F3’H, H^+^-ATPase and several TFs were selected from this QTL (Table 5). F3’H is an important enzyme in the pathway of anthocyanin synthesis whose expression shows an important influence on anthocyanin composition [2, 3, 58]. Anthocyanins accumulate in vacuoles, and the pH in the vacuoles has an important influence on the stability of anthocyanins and color development. H^+^-ATPase plays important roles in controlling vacuolar pH and anthocyanin transport [25]. Overall, in addition to the major color QTL on LG2, multiple QTLs were detected for anthocyanin content and composition. A series of candidate genes were selected from these QTLs. Increasing the size of the genetic population can effectively increase the exchange rate of genome segments, which can improve the precision of QTL mapping [59, 60]. In our study, the F1 population used for QTL mapping was relatively small. We can further improve the precision of QTL mapping by increasing the size of the population in future research.

## Conclusions

Berry color is an important trait for grapes and wine, and it is important to further explore the coloring mechanism of grapes. In this study, QTL mapping was performed based on a high-density genetic map for grape berry color. In addition to a major QTL col-2-1 on LG2 containing *MYBA* genes, multiple QTLs were found to be associated with berry color. In particular, another QTL, col-2-2, on LG2 and the QTL col-17-1 on LG17 showed very significant associations with anthocyanin content and composition. This strongly indicates that grape berry color is a quantitative trait controlled by multiple loci. After considering the gene annotation and RNA-seq data, several new candidate genes were selected from the above QTL regions. Although the function of these new candidate genes needs to be further verified, these results are of great significance for research on the regulatory mechanism of grape berry color.

## Methods

### Plant materials

*V. vinifera* cv. ‘Red Globe’ and ‘Muscat Hamburg’ and their 95 true hybrid progenies, which were confirmed using five SSR markers (VVMD27, VVMD28, Vchr3a, VMC1C10, VrZAG67), were used in this study. The plants were grown in the fields of the Pomology Institute, Shanxi Academy of Agricultural Sciences (37°23ˊN, 112°32ˊE). Rows in the north-south direction were used in the vineyard. We evaluated the coloration of the berries and detected anthocyanins in the berry skin when the grape berries were ripe. The method of observing the color of seeds and detecting the soluble solids was adopted to judge the ripening of berries. When the seeds were completely browned, and the soluble solids reached 18 or more on average and did not increase for a week, the berries were considered to be in the ripening stage.

The berry skins of parents were collected at 4 different fruit developmental stages: before veraison (40 DAF), at veraison (80 DAF), before the fruit ripening period (100 DAF) and during the fruit ripening period (120 DAF). At each stage, 3–5 clusters of ‘Red Globe’ and ‘Muscat Hamburg’ were harvested, and 20 berries were collected from each cluster. The skins of 40–50 berries were collected. Three biological replicates were performed at each stage of ‘Red Globe’ and ‘Muscat Hamburg’.

### DNA extraction, library construction and sequencing

The genomic DNA extraction kit (Tiangen Biotech, Beijing, China) was use to extract genomic DNA from young leaves of the parents and progeny. The quality of the DNA was detected on agarose gels. The enzyme-labeled instrument (Multiskan FC, Thermo Scientific™, Waltham, MA, USA) was used to measure DNA concentration. After DNA extraction, the Covaris was used to fragment the genomic DNA, followed by purification and repair. Then, the fragments were added Illumina adaptors. PCR was performed to enrich the fragments with the adaptors. After further quality control and purification, the libraries were subjected to 150-base paired-end sequencing using a Hiseq X Ten platform (Illumina, San Diego, CA, USA) by BGI Genomics Co., Ltd. (Shenzhen, China). The sequencing depths of the parents and offspring were 30X and 10X, respectively.

### Genotyping and genetic map construction

The grape PN40024 assembly 12X was used as reference genome [39]. The software BWA v0.7.15 was used to align clean reads to reference genome. GATK 3.7 was used to perform SNP detection, The SelectVariants and VariantFiltration tools of GATK 3.7 were used to filter the SNP markers (filtration parameters: QD < 2.0 / FS > 60.0 / MQ < / MQRankSum < 40.0–12.5 / ReadPosRankSum < 8.0). The obtained population SNPs were further filtered to remove reads with depth (DP) less than 10, quality value (GQ) less than 40, deletion rate more than 20% and non-dimorphic SNPs, so as to obtain high-quality population SNP markers. The LepMap3 was used to construct a genetic map based on SNP markers. The Filtering2 function of LepMap3 was used to filter the partial separation markers. The SeparateChromosomes2 function of LepMap3 was used to perform linkage group clustering, and an LOD score of ‘7’ was set as the threshold for determining whether loci were linked or not. SVG was used to draw the collinear graph to show the collinear relationship between genetic and physical map. Spearman correlation coefficients were also calculated to assess the collinearity between the genetic and physical maps.

### Evaluation of grape berry color

According to the coloration of the berries, the population was graded from 0 to 5. Grade 0 represents the white (yellow-green) color type, and grade 5 represents the purple-black color type. The grades from level 0 to level 5 indicate berry colors varying from light to dark. In addition, a colorimeter (CR-410, Konica Minolta Camera, Co, Ozaka, Japan) was also used to evaluate the coloration of the berry skin.

### Anthocyanin extraction and measurement

Extraction method of anthocyanins from grape berry skin was referred to He et al. [7] and Sun et al. [61, 62]. The UPLC-MS (Waters, Milford, MA, USA) was used to determine the composition and contents of anthocyanins, and the method was referred to Sun et al. [61].

### QTL analysis

MapQTL 6.0 was used to calculate marker cosegregation and QTL position. Phenotypic, map and loci information were imported into MapQTL 6.0. Multiple QTL mapping (MOM) methods were used to perform QTL analysis.

### RNA sequencing

Total RNA from grape berry skin was extracted using the TIANGEN plant RNA extraction Kit (Tiangen Biotech, Beijing, China). Then, the RNA concentration was measured using an enzyme-labeled instrument (Multiskan FC, Thermo Scientific™, Waltham, MA, USA). Then, the RNA sequencing was performed using BGISEQ-500 platform (BGI, Shenzhen, China).

### qRT-PCR analysis

In order to validate the results of RNA-seq, we selected 20 genes related to anthocyanin accumulation and designed primers (Table S6) to perform qRT-PCR. The qRT-PCR were performed with Roche LightCycler480 Real-Time Detection System (Roche). The *Actin* was used as the reference gene to calculate the relative expression of the target genes by 2^- ΔΔ CT^ method [63].

### Statistical analysis

The SPSS software (SPSS 22.0, Chicago, IL, USA) was used to perform statistical analysis. All experimental data used for comparative analysis were based on three biological replicates.

## Supplementary information

**Additional file 1: Figure S1.** The diagram of the anthocyanins biosynthetic pathway.

**Additional file 2: Figure S2.** The collinearity analysis between genetic and physical map.

**Additional file 3: Figure S3.** The QTL localization for total anthocyanins (TA) and the proportions of trihydroxylated anthocyanins (DC), methylated anthocyanins (MC) and acylated anthocyanins (AC) on LG4, LG6 and LG11.

**Additional file 4: Figure S4.** qRT-PCR validation of RNA-Seq data. Histograms represent expression levels as assessed by RNA-Seq, data are reported as means ± SE of 3 biological replicates (left axis). The line charts represent expression fold changes as assessed by qRT-PCR, data are reported as means ± SE of 3 replicates (right axis).

**Additional file 5: Table S1.** The statistics for the sequencing data and alignment with the reference genome.

**Additional file 6: Table S2.** The distribution of markers in the parents genetic maps.

**Additional file 7: Table S3.** The Spearman correlation coefficients between the genetic and physical map of each LG.

**Additional file 8: Table S4.** Mass spectrometry and chromatographic information for anthocyanins was detected in the population.

**Additional file 9: Table S5.** The expression levels and annotations of genes in the QTL regions.

**Additional file 10: Table S6.** Primers for qRT-PCR.

## Data Availability

All data generated or analysed during this study are included in this published article and its supplementary information files. The WGS and transcriptome data are available from SRA database of NCBI (Accession ID: PRJNA589353, PRJNA627817).

## Abbreviations

AC: Acylated anthocyanins
CA: Chromatic aberration
CG: Color grade
Chrs: Chromosomes
DC: Trihydroxylated anthocyanins
LGs: Linkage groups
MC: Methylated anthocyanins
QTL: Quantitative trait locus
SNPs: Single-nucleotide polymorphisms
TA: Total anthocyanins
TFs: Transcription factors
UFGT: UDP-glucose:anthocyanidin:flavonoid glucosyltransferase
WGS: Whole-genome resequencing
