# Supplementary figures and images for: New quantitative trait locus (QTLs) and candidate genes associated with the grape berry color trait identified based on a high-density genetic map
Source: BMC Plant Biol. 2020 Jun 30;20:302. doi: 10.1186/s12870-020-02517-x (PMC7325011; doi:10.1186/s12870-020-02517-x)

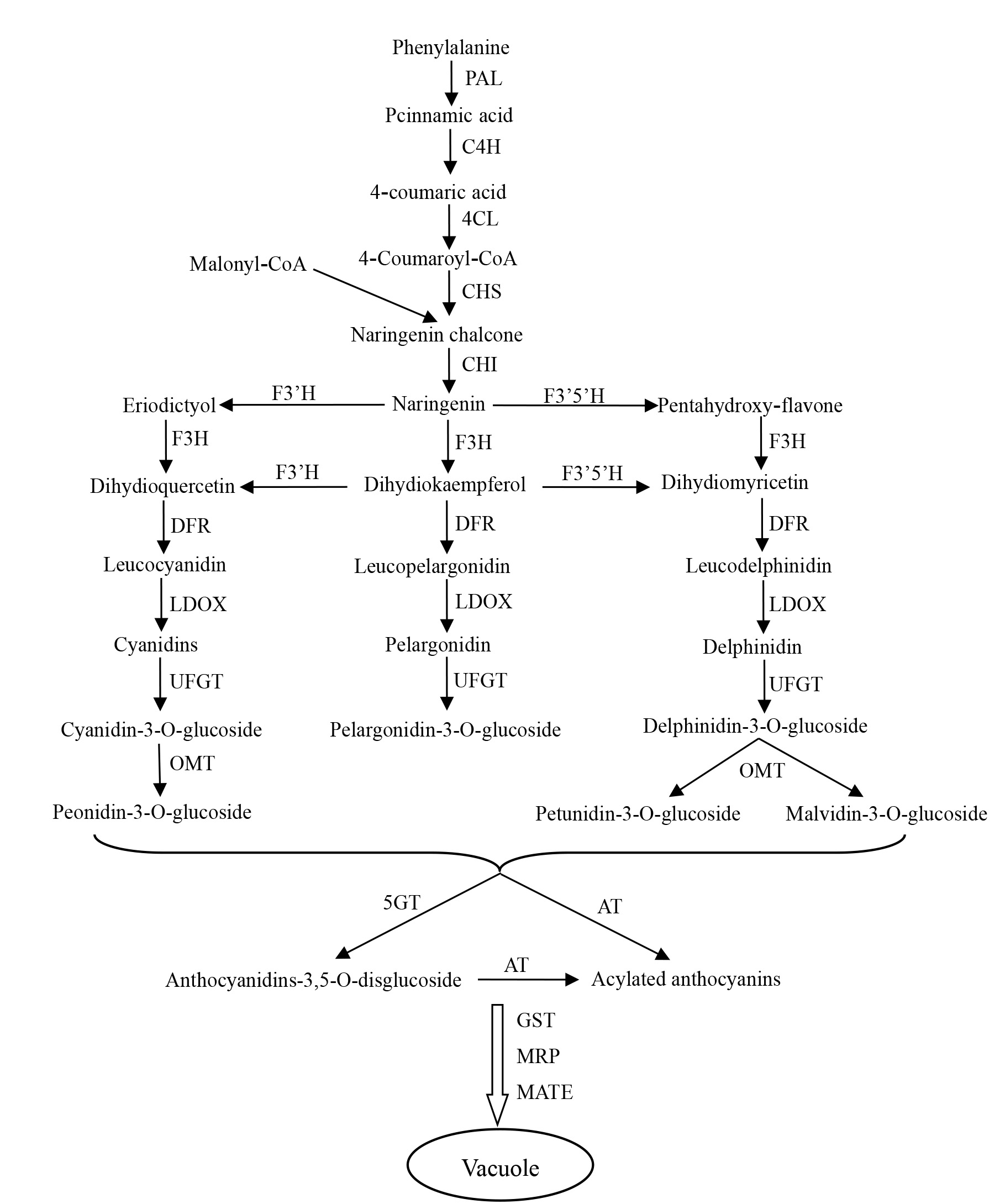

Supplement: Supplementary file 1 — Additional file 1: Figure S1. The diagram of the anthocyanins biosynthetic pathway. [file 12870_2020_2517_MOESM1_ESM.jpg]

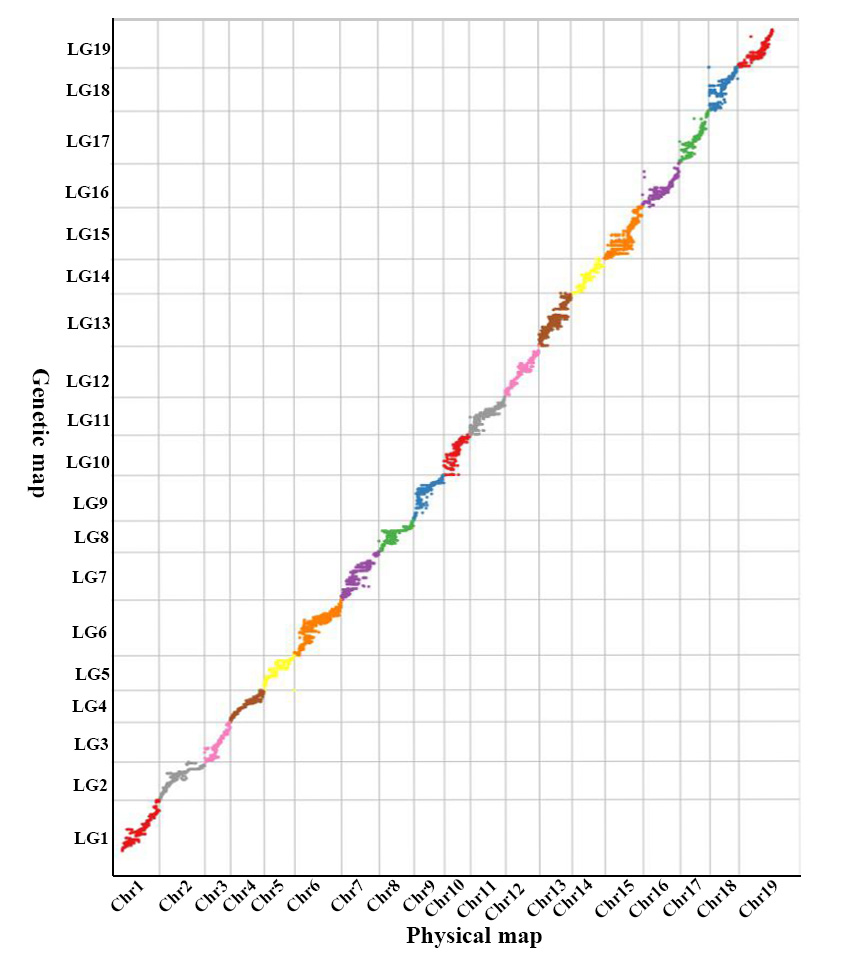

Supplement: Supplementary file 2 — Additional file 2: Figure S2. The collinearity analysis between genetic and physical map. [file 12870_2020_2517_MOESM2_ESM.jpg]

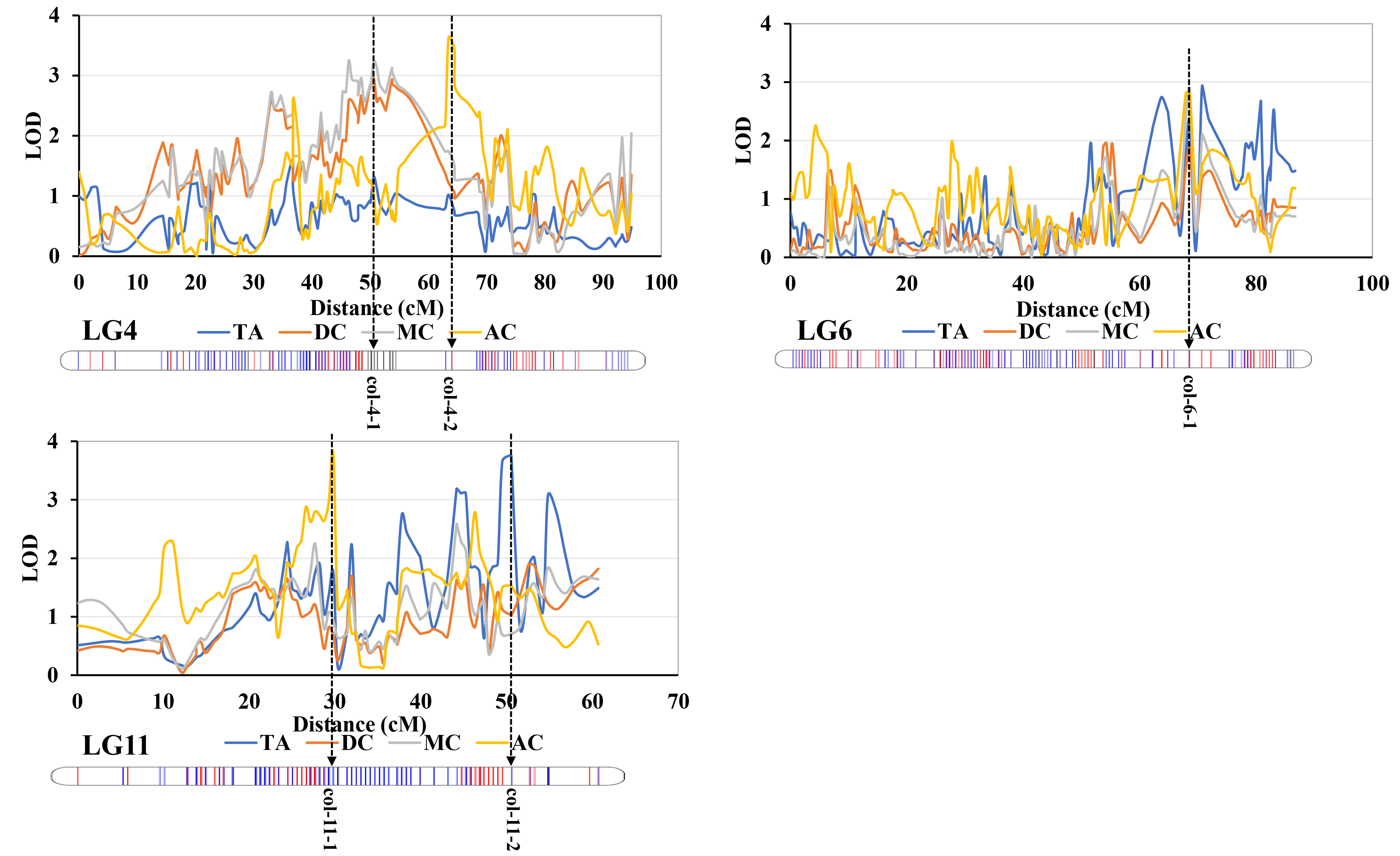

Supplement: Supplementary file 3 — Additional file 3: Figure S3. The QTL localization for total anthocyanins (TA) and the proportions of trihydroxylated anthocyanins (DC), methylated anthocyanins (MC) and acylated anthocyanins (AC) on LG4, LG6 and LG11. [file 12870_2020_2517_MOESM3_ESM.jpg]

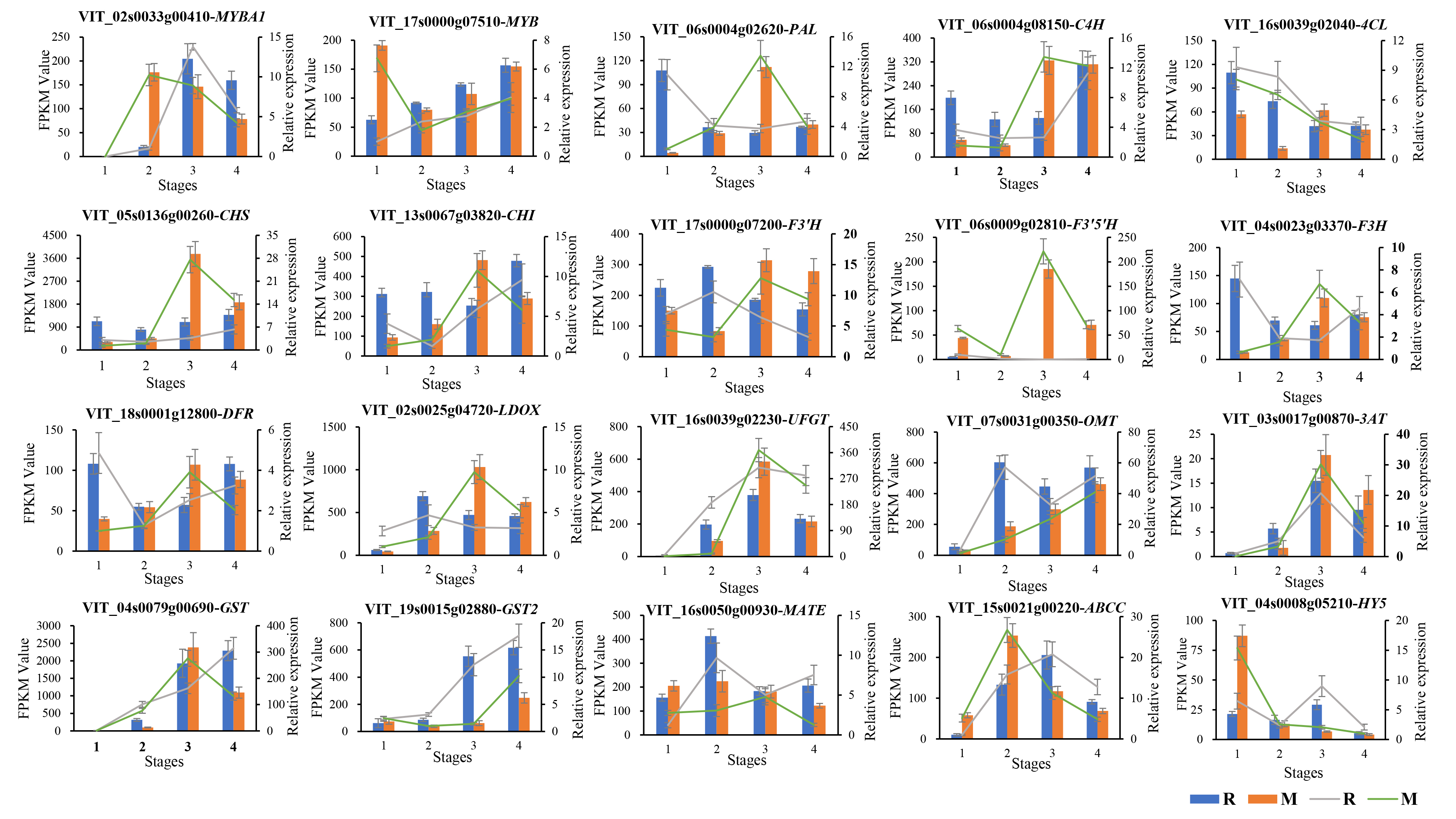

Supplement: Supplementary file 4 — Additional file 4: Figure S4. qRT-PCR validation of RNA-Seq data. Histograms represent expression levels as assessed by RNA-Seq, data are reported as means ± SE of 3 biological replicates (left axis). The line charts represent expression fold changes as assessed by qRT-PCR, data are reported as means ± SE of 3 replicates (right axis). [file 12870_2020_2517_MOESM4_ESM.jpg]
